# Temporal Alterations in Cerebrovascular Glycocalyx and Cerebral Blood Flow after Exposure to a High-Intensity Blast in Rats

**DOI:** 10.3390/ijms25073580

**Published:** 2024-03-22

**Authors:** Ye Chen, Ming Gu, Jacob Patterson, Ruixuan Zhang, Jonathan K. Statz, Eileen Reed, Rania Abutarboush, Stephen T. Ahlers, Usmah Kawoos

**Affiliations:** 1 Naval Medical Research Command, Silver Spring, MD 20910, USA; ye.chen.ctr@health.mil (Y.C.); ming.gu.ctr@health.mil (M.G.);; 2 The Henry M. Jackson Foundation for the Advancement of Military Medicine, Inc., Bethesda, MD 20817, USA; 3 Parsons Corporation, Columbia, MD 21046, USA

**Keywords:** blast, traumatic brain injury, cerebrovascular glycocalyx, cerebral blood flow, vascular pathology

## Abstract

The glycocalyx is a proteoglycan–glycoprotein structure lining the luminal surface of the vascular endothelium and is susceptible to damage due to blast overpressure (BOP) exposure. The glycocalyx is essential in maintaining the structural and functional integrity of the vasculature and regulation of cerebral blood flow (CBF). Assessment of alterations in the density of the glycocalyx; its components (heparan sulphate proteoglycan (HSPG/syndecan-2), heparan sulphate (HS), and chondroitin sulphate (CS)); CBF; and the effect of hypercapnia on CBF was conducted at 2–3 h, 1, 3, 14, and 28 days after a high-intensity (18.9 PSI/131 kPa peak pressure, 10.95 ms duration, and 70.26 PSI·ms/484.42 kPa·ms impulse) BOP exposure in rats. A significant reduction in the density of the glycocalyx was observed 2–3 h, 1-, and 3 days after the blast exposure. The glycocalyx recovered by 28 days after exposure and was associated with an increase in HS (14 and 28 days) and in HSPG/syndecan-2 and CS (28 days) in the frontal cortex. In separate experiments, we observed significant decreases in CBF and a diminished response to hypercapnia at all time points with some recovery at 3 days. Given the role of the glycocalyx in regulating physiological function of the cerebral vasculature, damage to the glycocalyx after BOP exposure may result in the onset of pathogenesis and progression of cerebrovascular dysfunction leading to neuropathology.

## 1. Introduction

Exposure to blast is a prevalent occurrence in the military during training or combat scenarios. Notable cerebrovascular complications were observed among combat casualties with severe blast-related traumatic brain injury (bTBI) during Afghanistan and Iraq wars [1,2,3]. Preclinical studies have shown that bTBI is associated with alteration in the cerebral vasculature [4,5,6,7,8,9,10]. The pathological underpinnings of such alterations may trigger a cascade of processes leading to chronic changes manifesting into cerebrovascular alterations and associated neurological deficits [5,9,11,12,13,14,15,16]. The microstructure of the cerebral vessels is vulnerable to damage due to the initial transfer of kinetic energy from blast waves to the brain either directly or via propagation through the vascular system [3,17].

The glycocalyx is a delicate grass-like microstructure lining the luminal surface of the vascular endothelium, which plays a pivotal role in maintaining vascular structure and function [18,19,20]. The structure of the glycocalyx mainly consists of proteoglycans and glycoproteins, which form the backbone of the glycocalyx [21,22]. The proteoglycans and glycoproteins have core proteins that form a structural bridge between the endothelium and glycosaminoglycan side chains which extend into the vascular lumen. The main proteoglycan core proteins are members of the syndecan (SD) and glypican family. The glycosaminoglycans that render the glycocalyx grass-like in appearance are predominantly heparan sulfate (HS), chondroitin sulfate (CS), dermatan sulfate, keratan sulfate, and hyaluronan or hyaluronic acid (HA). Among the core protein SDs, SD-1 and SD-2 are the major SDs adhering to the vascular endothelium [23]. Both SD-1 and SD-2 bind with HS and CS; thus, the integrity of these SDs significantly affects the structural stability of the glycocalyx. The most abundant glycosaminoglycan side chain is HS (50–90%) [24] followed by CS and dermatan sulphate (~12–20%) [25].

The glycocalyx is a multifunctional dynamic structure that acts as a barrier between the endothelium and plasma and has a significant role in maintaining vascular function [26,27]. By acting as a natural barrier for endothelial cells, the glycocalyx reduces the risk of damage to the endothelium by harmful components that may be circulating in the blood [27]. The glycocalyx regulates vascular permeability and blood–brain barrier (BBB) function [22,28,29]; hemodynamic properties by modulating blood viscosity [30,31]; vascular mechanoreceptor properties [32]; vascular tone [33]; inflammatory response [34]; and anticoagulative function [22]. The vascular endothelium autoregulates blood flow and the cerebrovascular glycocalyx is involved in various control processes that maintain cerebral blood flow (CBF) [18,35]. The glycocalyx is an important mechanosensor that transduces shear stress or forces induced by blood flow into chemical and intracellular signals, mediating vascular contractility and hydraulic conductivity [26]. HS, HA, and SDs (SD-1 and SD-2) are the main components of the glycocalyx that induce endothelial nitric oxide synthase production and resultant vasodilation in response to shear forces [22]. Barrier properties of the glycocalyx prevent adhesion of thrombocytes to procoagulant peptides on the endothelium, preventing accumulation of thrombocytes [21]. Additionally, anticoagulant molecules (such as HS) [36] present in the glycocalyx contribute to anticoagulatory properties of the structure [37] and support maintenance of blood viscosity and adequate CBF. Degradation of the glycocalyx allows for ease of blood cell–endothelium interactions and an increase in the availability of procoagulant molecules, resulting in acute coagulopathy [38,39]. Thus, a healthy glycocalyx is an important contributor towards maintaining homeostasis in the vascular microenvironment.

The glycocalyx serves as a major line of defense against endotheliopathy in brain trauma [27]. Thinning of the glycocalyx layer and shedding of its components are present in pathological sequelae of traumatic brain injury (TBI) leading to endothelial disruption and its consequent downstream effects [26,40,41]. Owing to the role of the glycocalyx in regulation of various vascular functions, its attenuation in the cerebral vessels is associated with processes that include changes in the BBB and vascular permeability [42]; vascular tone [43]; and hemodynamics [27]. The breakdown of the glycocalyx assessed by an increase in shedding of SD-1 was associated with subsequent coagulopathy and poor outcome after 72 h in severe TBI patients [40]. Exposures to twelve relatively low-level (e.g., 5.8 PSI/40 kPa) repeated blast overpressure (BOP) events over a prolonged period were shown to produce broad degradation of the glycocalyx in multiple brain regions in a rodent model [41]. However, the effect of a single high-intensity blast exposure on the cerebrovascular glycocalyx remains to be fully understood. Given that brain injury is associated with perturbations of the glycocalyx in the cerebral vessels, alterations in CBF and tissue perfusion after injury are some of the probable outcomes. Vascular disruptions due to TBI contribute to CBF dysfunction [44,45] causing a reduction in CBF and delivery of oxygen and metabolites to the brain [46,47,48,49]. Decreased CBF has been linked to disruption of autoregulation in the microvasculature following brain injury [50,51]. Subtle reductions in CBF that may not be low enough to induce an ischemic state can exacerbate injury due to dampened tissue perfusion and hypoxia. Both hypoxia and ischemia have been shown to initiate degradation of the glycocalyx [52]. The changes in the glycocalyx and CBF appear to have an interdependent role in determining the pathological trajectory of TBI.

The relevance of the glycocalyx regarding vascular morphology and function, which are altered by exposure to blast, makes further assessment of blast-related changes to the glycocalyx critical in understanding the mechanisms of vascular changes and deficits in bTBI. The study presented here was based on the hypothesis that exposure to a high-intensity blast induces time-dependent alterations to the cerebrovascular glycocalyx and CBF. This study assesses temporal alterations in the density of the glycocalyx; its components (heparan sulphate proteoglycan (HSPG/SD-2), HS, and CS); CBF; and the responsiveness of CBF to stimulus in the form of hypercapnia after a single high-intensity BOP exposure.

## 2. Results

### 2.1. Shedding of Endothelial Glycocalyx Induced by Blast Exposure Followed by Recovery over Time

**Figure 1:**
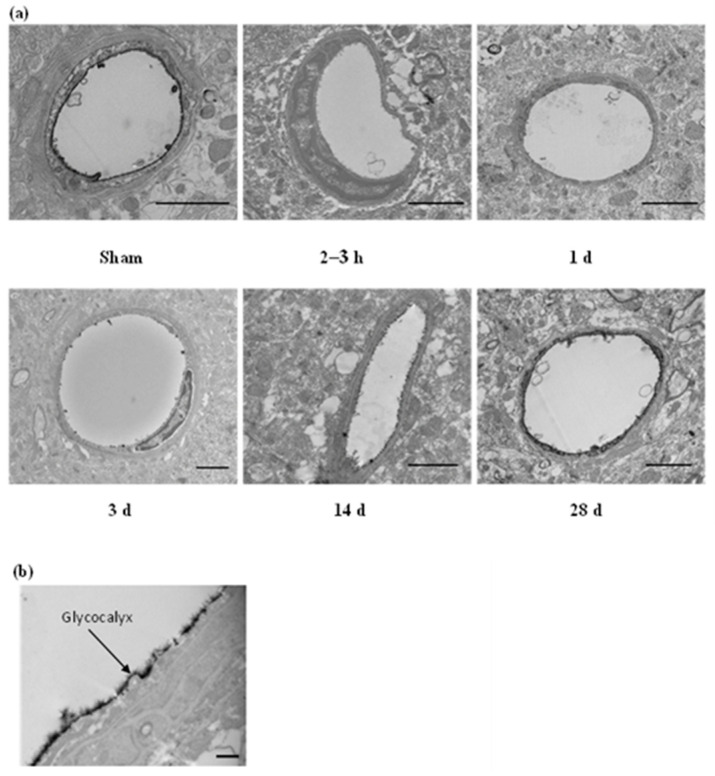
(**a**) Electron micrographs showing representative cerebral vessels from sham and blast groups. Glycocalyx is seen as the dark bushy layer lining the vascular lumen in sham-exposed animals. Depletion of glycocalyx is observed in BOP-exposed animals with partial (14 days) and full (28 days) recovery over time. Scale bar: 2 µm. (**b**) High-magnification (25,000×) micrograph showing grass-like appearance of glycocalyx. Scale bar: 500 nm. h: hours, d: days.

Electron micrographs indicated that blast exposure resulted in ultrastructural alterations in the cerebral vasculature acutely and chronically. Figure 1a presents representative micrographs of vessels from sham- and BOP-exposed groups; and Figure 1b is a high-magnification micrograph of the glycocalyx, showing its grass-like appearance. The endothelial glycocalyx was observed as a thick, even, and continuous layer covering the luminal surface of the endothelium in sham-exposed rats. However, vessels imaged from BOP-exposed animals showed degradation of the glycocalyx, which included reductions in the density and continuity of the glycocalyx. Less than 5% of the randomly selected micrographs of capillaries were found to lack a glycocalyx in the sham group, while approximately 35% of the imaged capillaries in the acute phase (2–3 h post-BOP exposure) showed a complete absence of the glycocalyx, suggesting BOP exposure may result in mechanical cleaving of the glycocalyx. At later post-blast time points, recovery of the glycocalyx was observed in only 14% of vessels at 3 d, and 4% at 14 and 28 days showed a complete lack of the glycocalyx. To further examine the effects of BOP exposure on the endothelial glycocalyx, parameters describing glycocalyx morphological changes including (1) the distribution of the glycocalyx present along the vascular lumen (glycocalyx percentage), (2) the thickness of the glycocalyx, and (3) the G-index, a metric to quantify changes in the thickness of the glycocalyx and endothelial ‘thinning’ with respect to the size of a vessel, were estimated and compared between sham- and BOP-exposed groups.

### 2.2. The Distribution and Thickness of Glycocalyx along the Vascular Lumen Were Significantly Decreased by Blast Exposure Followed by Recovery over Time

**Figure 2:**
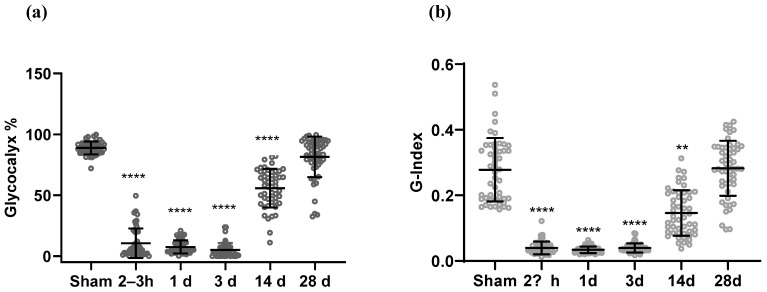
Semi-quantitative analysis of endothelial glycocalyx and the temporal effect of blast exposure on its depletion and recovery. (**a**) Glycocalyx % is the percentage of vascular lumen covered with glycocalyx. (**b**) G-index is a measure that accounts for thinning of glycocalyx and endothelium with respect to vessel diameter, ** *p* = 0.0037, **** *p* < 0.0001 when compared with sham. h: hours, d: days.

Electron micrographs of approximately 60 vessels (30 vessels/animal; lumen diameters ranging from 3 µm to 7 µm) from the frontal cortex in sham- and BOP-exposed groups were randomly selected and analyzed for glycocalyx percentage, thickness, and G-index. The mean ± standard deviation of lumen diameters for the groups were as follows: sham, (5.34 ± 1.43) µm; 2–3 h, (5.55 ± 1.5) µm; 1 day, (5.67 ± 1.41) µm; 3 days, (5.13 ±1.46) µm; 14 days, (5.25 ± 1.31) µm; and 28 days, (5.51 ± 1.51) µm. Figure 2a presents the percentage of vascular lumen covered by glycocalyx in each group. In sham animals, 80.80 ± 3.30% (mean ± standard deviation) of the vascular lumen was covered by the glycocalyx (glycocalyx percentage), which was significantly higher than that in animals at 2–3 h and 3 and 14 days post-BOP exposure (*p* < 0.0001 for blast groups versus sham, Table 1). By 28 days, glycocalyx percentage in BOP-exposed rats showed full recovery to match the sham controls. The thickness of the glycocalyx was measured and used to calculate the G-index (Figure 2b). Briefly, significant reduction in the thickness of the glycocalyx was observed at 2–3 h (0.05 ± 0.091 nm), 1 day (0.027 ± 0.005 nm), and 3 days (0.028 ± 0.008 nm) in comparison with sham (1.156 ± 0.06 nm). Recovery in the thickness of the glycocalyx in the BOP-exposed animals was observed starting at 14 days (0.58 ± 0.053 nm); and the thickness at 28 days (1.267 ±0.067 nm) slightly surpassed that of the sham group. Descriptive statistics are presented in Table 1. A similar trend was observed in the G-index (Figure 2b), which showed significant reduction at 2–3 h and 1 and 3 days (*p* < 0.0001), partial recovery at 14 days (*p* = 0.0041) and full recovery at 28 days when compared with the sham group. The return of the G-index to control levels by 28 days is indicative of repair in both the glycocalyx and the endothelium.

### 2.3. Blast Exposure Led to Alteration in the Concentration of Glycocalyx Components in Brain and Plasma

**Figure 3:**
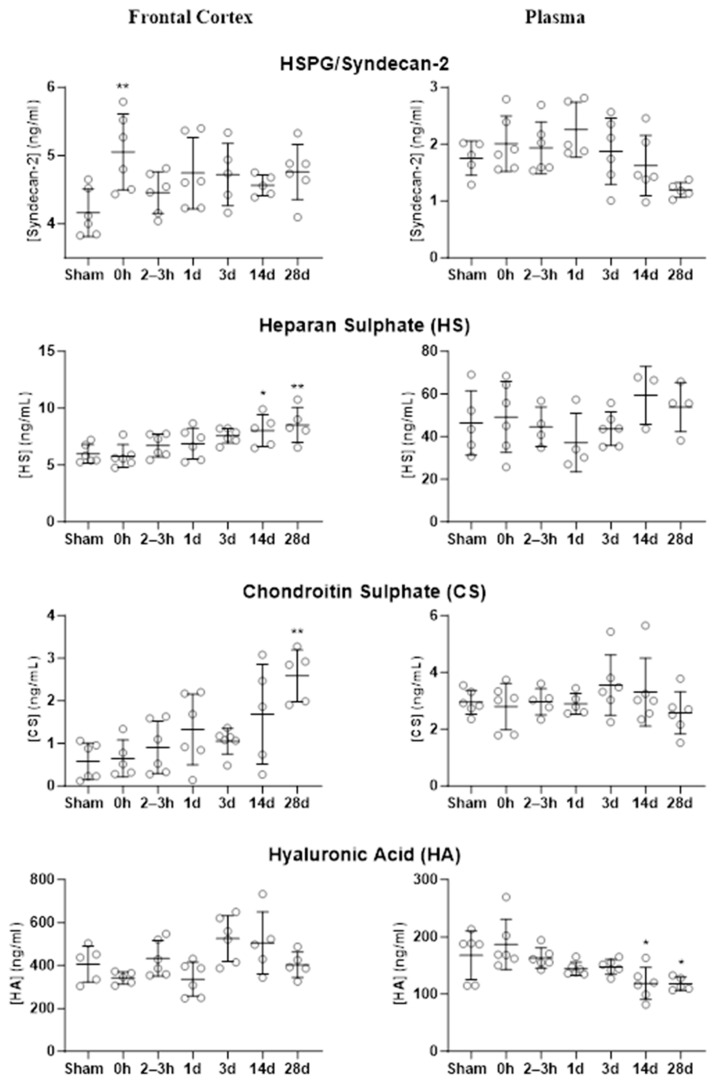
Concentration of glycocalyx components in the frontal cortex and plasma at pre-determined post-blast time points and their comparison with sham; 0 h is immediately after BOP exposure. * *p* = 0.05, ** *p* < 0.01. h: hours, d: days.

The concentrations of four components of glycocalyx (SD-2, HS, CS, and HA) were assessed in the frontal cortex and plasma (Figure 3). Immediately after BOP exposure, a significant increase in SD-2 was observed in the frontal cortex (*p* = 0.0041, 0 h i.e., immediately after blast, compared with sham), and the levels remained elevated at all post-blast time points. This pattern was not observed in plasma. Though not statistically significant, SD-2 in plasma trended upwards after BOP exposure and was followed by a reduction at the later time points. HS and CS concentrations also trended upwards in the frontal cortex, reaching statistical significance at 14 days (HS) and 28 days (HS and CS) after BOP exposure. In the frontal cortex, levels of HA at 3 and 14 days were elevated (overall *p*-value of 0.0074 but no statistical significance when compared with sham); and plasma levels of HA showed a significant decrease at 14 and 28 days (*p* < 0.05). Overall, the levels of glycocalyx components in the frontal cortex after a single high-intensity BOP exposure showed sustained elevations. By 28 days, the levels of glycocalyx components in the plasma were comparable to sham levels except for HA, which was significantly reduced at 14 and 28 days. The results of statistical analyses are presented in Table 1.

### 2.4. Blast Exposure Induced Changes in Cerebral Blood Flow and Response to Hypercapnia

**Figure 4:**
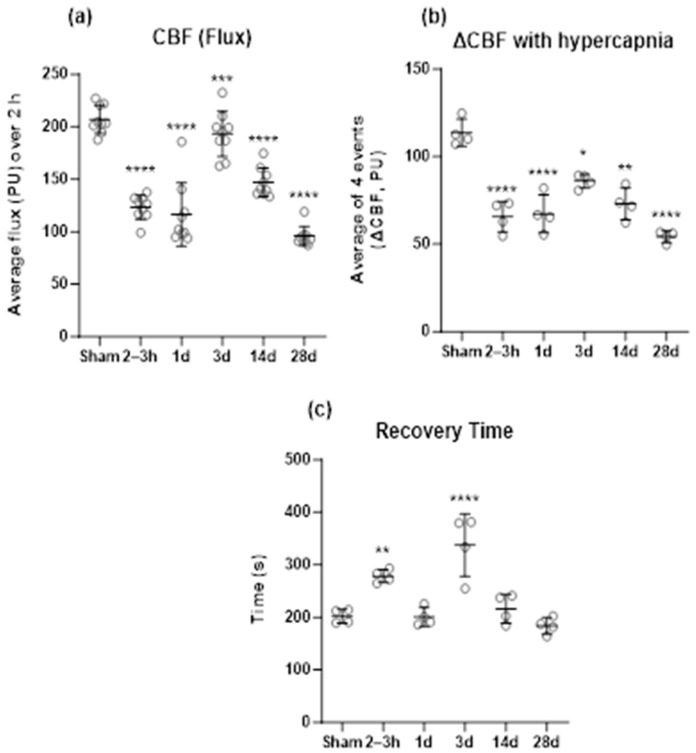
Effect of BOP exposure on (**a**) cerebral blood flow (CBF, measured as flux and expressed in perfusion units, PU); (**b**) change in CBF (ΔCBF) due to hypercapnia; and (**c**) the recovery time for CBF to return to pre-hypercapnia levels after each episode of hypercapnia. CBF for each animal is presented as an average value for the duration of a two-hour observation period. * *p* < 0.05, ** *p* < 0.01, *** *p* < 0.001, **** *p* < 0.0001. h: hours, d: days.

BOP exposure led to a sustained decrease in CBF (flux) and a diminished response to hypercapnia (Figure 4). Interestingly, the alterations in CBF (Figure 4a) and response to hypercapnia (Figure 4b) in BOP-exposed animals presented a biphasic trend with initial post-blast reduction (2–3 h, 1 day), some recovery at 3 days, followed by a second reduction (14 days, 28 days). Both CBF and change in CBF due to hypercapnia were at their lowest at 28 days after BOP exposure. At each hypercapnia event, the recovery time (latency in recovery of CBF to pre-hypercapnia levels after cessation of stimulation, Figure 4c) was elongated at 2–3 h and 3 days post-blast in comparison to the sham group, with the recovery time being delayed the most at 3 days post-blast. The results of statistical analyses are presented in Table 2.

## 3. Discussion

BOP exposure subjects the vascular hydrodynamic system to a surge in pressure that is orders of magnitude higher than the steady state. The glycocalyx is a delicate structure on the vascular endothelial cells, sensitive to changes in the vascular environment that can lead to its degradation followed by endogenous regeneration [53]. To date, only one study (Hall et al. [41]) has documented the effects of repeated low-level BOP exposures on the cerebrovascular glycocalyx. The investigation presented here is the first to demonstrate the time course of depletion and recovery of the cerebrovascular glycocalyx after exposure to a high-intensity blast. This work also assessed time-matched disturbances in CBF and the ability of the cerebrovasculature to chemoregulate as measured by the effect of hypercapnia on CBF. Briefly, the findings were as follows: significant depletion of the glycocalyx observed acutely (2–3 h) and 1 and 3 days after BOP exposure; recovery of the glycocalyx by 28 days after the exposure which was associated with an increase in HS (14 and 28 days) and increases in HSPG/SD-2 and CS (28 days) in the frontal cortex; and a significant decrease in CBF and a diminished response to hypercapnia at all time points with some recovery at 3 days post-blast. Previous studies using the same high-intensity blast model and post-blast time points for assessment of cerebrovascular changes have reported significant increases in BBB permeability at 2–3 h and 1 day after BOP exposure (Kawoos et al. [7]) and alterations in vascular reactivity of pial vessels to hypercapnia (Abutarboush et al. [4]). Notably, the expected vasodilatory response to hypercapnia was amplified (3–4 times that of sham controls) in small vessels (<50 µm) at 3 and 28 days post-blast but was impaired in medium-sized vessels (50–100 µm) at all time points except at 14 days where significant vasoconstriction was observed. The amplified vasodilatory response at 3 days post-blast could be a possible contributor to the partial recovery of CBF and change in CBF due to hypercapnia at the same post-blast time point (Figure 4a,b). Similarly, the switching of vascular reactivity of medium vessels from a dilatory to constrictive state at 14 days and a diminished dilatory response at 28 days are indicative of impaired vascular tone after BOP exposure, which may have a potential role in further reduction of CBF at 14 and 28 days as observed in this study. Collectively, the findings are suggestive of BOP exposure resulting in profound and enduring changes in the cerebral vascular structure and impairment of regulatory mechanisms portending the development of cerebrovascular pathologies.

The endothelial glycocalyx is a mechanosensor that modulates vascular response to mechanical forces such as shear stress from flowing blood; transduces the force to a biochemical response to maintain vascular tone; and plays a role in preventing coagulation, characteristics that facilitate regulation of CBF. The glycocalyx, by virtue of being a physical electrostatic barrier (due to negative charges on components like HA), prevents plasma proteins and negatively charged cells from attaching to the endothelium, thus averting perturbances in blood flow [29,54,55]. There is emerging evidence that components of the glycocalyx (HS and SD-1) bind with anticoagulant molecules [19,56] that trigger multistep processes which eventually inhibit platelet aggregation and cause relaxation of smooth muscles and vasodilation [57]. Our study showed complete cleavage of the glycocalyx in the acute phase after blast exposure and a significant reduction in CBF. The findings from this study also presented a significant recovery of the glycocalyx and an increase in HS in the brain which corresponded with previously reported enhancement of vasodilatory properties of small pial arterioles [4] at 28 days post-blast. Additionally, increases in the components of glycocalyx (SD-2, HS, CS, HA) in the brain were observed for up to 28 days after blast exposure. Such abundance of glycocalyx constituents may be due to compensatory mechanisms causing an over-production of the deficient components of the glycocalyx to reinstate its normal state. Plasma levels of glycocalyx components (though not specific to the cerebrovascular glycocalyx) have shown association with clinical outcomes after trauma [38,58,59], TBI [40,60], subarachnoid hemorrhage [61], and ischemic stroke [62,63,64,65]. Trauma is associated with elevation in the circulating levels of HA, HS, CS, and SD-1 [38]. Increases in the levels of SD-1 showed negative correlation with survival outcome after trauma and TBI [40,59,60]. Patients with ischemic stroke had an increase in the levels of CS, HS, SD-3 and a decrease in SD-2 in plasma at 7 days after the stroke, which returned to pre-stroke levels at 90 days [64]. Though not statistically significant, we found elevated levels of SD-2 in plasma at 2–3 h and 1 day after BOP exposure and electron microscopy (EM) showed significant shedding of the glycocalyx at the same time points. At 14 and 28 days post-blast, statistically significant reduction in plasma levels of HA was complementary to recovery of the glycocalyx observed via EM. The circulating levels of glycocalyx components appear to be predictive of clinical outcome after an adverse event and, thus, can potentially be used as biomarkers of injury and prognosis. The glycocalyx is a remarkable clinical target due to its functional role at the core of cellular events in both health and disease. Our findings on the depletion and regeneration of the cerebrovascular glycocalyx and alterations in its circulating components after bTBI have the translational potential for being implemented in clinical research and practices.

Among the markers of injury severity and predictors of prognosis after TBI, alterations in CBF (which have deleterious effects on cerebrovascular health) have been reported after TBI. Our results show reductions in CBF in the acute and chronic phase after blast exposure. Though some recovery is observed at 3 days after BOP exposure, a persistent reduction in CBF is seen for up to 28 days. Several other studies have reported short- and long-term effects of blast exposure or TBI on CBF. In mild TBI patients, reduced CBF in the thalamus was observed at one and nine months [66] and approximately two years after injury [67]. Reductions in CBF and changes in perfusion have shown a strong correlation with neuropsychological and cognitive impairment over extended periods of time after TBI or blast exposure [14,67]. Low CBF was also associated with worsened pathological outcome and high incidence of mortality; and recovery in CBF improved the outcome after TBI [68]. Blast exposure(s) during military training operations and/or combat-related TBI trigger a cascade of pathophysiological processes, even in the absence of overt injury, that determine the trajectory of brain health. In veterans with bTBI, the mean global CBF was significantly lower at three years after injury when compared to age-matched veteran controls [69]. Blast exposure was shown to induce up to 27% reduction in CBF in the hippocampus in animal studies [70] and reduced CBF along with association between a reduction in CBF in the cingulate cortex and depletion of white matter integrity in the cingulum bundle in veterans [71] during the chronic phase after TBI. Emerging evidence shows that blast exposure(s) are associated with acute and chronic structural and functional disruption to the cerebral vasculature [4,7,11,13,72,73,74], which contribute to the course of changes in CBF and pathological trajectory of TBI.

Taken together, the data presented here demonstrate that an exposure to a single high-intensity BOP leads to temporal alterations in the cerebrovascular glycocalyx. Significant reduction in CBF and responsiveness of CBF to hypercapnia were a sustained deficit after BOP exposure. This study elucidates possible molecular mechanisms associated with alterations in the cerebral vasculature and sustained deficits observed after exposure to a single high-intensity blast. However, functional manifestations of such alterations in the form of motor or cognitive performance were not addressed, which is a limitation of this study. There is a paucity of data on the changes in the glycocalyx and associated effect on CBF after exposures to repeated low-level blasts. Repetitive blasts and subconcussive impacts are of increasing interest as potential risk factors contributing to chronic neuropathologies and their manifestations observed in military personnel and athletes with a history of repeated exposures or injuries [75,76,77]. Alterations in the glycocalyx and CBF and the potential of circulating constituents of the glycocalyx as biomarkers of bTBI due to repeated blast exposures warrant further investigation.

## 4. Materials and Methods

### 4.1. Blast Exposure

Calibrated experimental blast exposure was performed as described previously [7,78]. Briefly, an air-driven blast simulator composed of a compression chamber and an expansion chamber was used to deliver blast exposure. The two chambers were separated by a polyethylene terephthalate, Mylar, membrane (Tekra, LLC., New Berlin, WI, USA), and the thickness of the membrane determined blast wave characteristics. Anesthetized animals (5% isoflurane for 2.5 min) were placed in a restrainer located at the open end of the expansion chamber. To restrict head and body movements caused by the blast wave, rats were placed in a plastic cone (DeCapi Cones; BrainTree Scientific Inc., Braintree, MA, USA) and secured by three rubber tourniquets crossing the head, chest, and lower torso in the restrainer. All animals in blast exposure groups received a single blast head on with their bodies positioned in line with the direction of blast wave propagation. After blast exposure, rats were allowed to recover from anesthesia and returned to their cages until the appropriate study time point. The blast wave parameters expressed as mean ± standard deviation for the positive phase (overpressure) were as follows: peak pressure, 18.9  ±  0.25 PSI (131 ± 1.72 kPa); duration, 10.95  ±  0.99 ms; impulse, 70.26  ±  1.18 PSI·ms (484.42 ± 8.14 kPa·ms); and those of the negative phase (underpressure) were as follows: peak magnitude of 1.28  ±  0.20 PSI (8.83 ± 1.38 kPa), duration of 11.3  ±  0.91 ms, and impulse of 3.74  ±  0.47 PSI·ms (25.79 ± 3.24 kPa·ms), as described previously [7].

### 4.2. Animals

The study protocol was reviewed and approved by the Walter Reed Army Institute of Research (WRAIR)/Naval Medical Research Command (NMRC) Institutional Animal Care and Use Committee in compliance with all applicable federal regulations governing the protection of animals in research. The experiments reported herein were conducted in compliance with the Animal Welfare Act and per the principles set forth in the “Guide for Care and Use of Laboratory Animals”, Institute of Laboratory Animals Resources, National Research Council, National Academy Press, 2011. The experiments are reported in compliance with ARRIVE guidelines 2.0 [79]. Male Long Evans rats weighting 300–350 g (Charles River Laboratories, Wilmington, MA, USA) were randomly distributed into blast groups over post-blast time points of 0 h (hours, 2–9 min after blast), 2–3 h, 1, 3, 14, or 28 d (days) and a sham group. The sham control group was treated like the blast groups except for exposure to blast. Sham-exposed rats were euthanized at 2–3 h after undergoing sham exposure procedures.

### 4.3. Experimental Design

The study was conducted under two experiments.

Experiment 1: Assessment of blast-induced ultrastructural alterations in (1) the glycocalyx using electron microscopy (EM); and (2) concentrations of structural components of the glycocalyx in the brain and plasma using enzyme-linked immunosorbent assay (ELISA) was performed. Separate groups of animals (*n* = 3 for EM and *n* = 6 for ELISA) were used for each sub-experiment and post-blast time point. A group of animals (0 h, *n* = 6) were euthanized immediately after exposure to blast to assess the mechanical effects of blast on shedding of the glycocalyx using ELISA.

Experiment 2: The effect of blast on CBF and the alterations in CBF induced by hypercapnia in sham- (*n* = 8) and blast-exposed groups (*n* = 6–8/group) were evaluated.

### 4.4. Electron Microscopy

Animals were administered an intraperitoneal injection of Euthasol (80–100 mg/kg, pentobarbital sodium and phenytoin sodium) and after the cessation of response to toe pinch, they were transcardially perfused with 0.9% saline followed by fixation buffer which was administered in two steps. The first step was perfusion with approximately 30 mL of fixative solution containing 4% paraformaldehyde, 1% glutaraldehyde, and 30 mMol/L magnesium chloride prepared in 1X phosphate-buffered saline (PBS). The second step followed immediately by perfusion with 500 mL of fixative solution with 0.05% (*w*/*v*) Alcian Blue 8GX (Electron Microscopy Sciences, Hatfield, PA, USA) added to the solution from the previous step. The brains were harvested and a 1 mm biopsy punch was used to extract samples from the frontal cortex which were post-fixed in fixative solution containing Alcian Blue for at least 72 h. The procedure described by Hall et al. [41] was used to process the post-fixed samples for EM scanning. Briefly, the samples were rinsed with 1X PBS for 5 min, followed by fixation with 1% osmium tetroxide (Electron Microscopy Sciences, Hatfield, PA, USA) and contrast staining with 1% uranyl acetate (Electron Microscopy Sciences, Hatfield, PA, USA) for one hour each. Sections were then dehydrated by sequential concentrations of ethanol (50%, 70%, 90%, 100%) (Sigma, St. Louis, MO, USA) and propylene oxide (Electron Microscopy Sciences, Hatfield, PA, USA). Sections were embedded in Epon 812 acrylic resin (Electron Microscopy Sciences, Hatfield, PA, USA) and ultrathin sections of tissue (50 nm) were placed on copper grids for staining with 2% uranyl acetate (Electron Microscopy Sciences, Hatfield, PA, USA) for 30 min, followed by rinsing with distilled water and staining with lead citrate (Electron Microscopy Sciences, Hatfield, PA, USA) for 2 min. Ultrastructural examination of the stained ultrathin tissue sections and micrograph acquisition were performed by a transmission electron microscope (EM-21010, JEOL Ltd., Peabody, MA, USA).

### 4.5. Micrograph Analysis of Glycocalyx

Semi-quantitative analyses of the glycocalyx were carried out using ImageJ which is an open-source image processing program developed by National Institutes of Health, Bethesda, MD. Two metrics were used to quantify the glycocalyx: (1) glycocalyx percentage (the percentage of vascular lumen that was covered with glycocalyx) and (2) G-index (ratio of the combined thickness of glycocalyx and endothelium to diameter of the vessel’s lumen). To calculate the G-index, eight equally spaced regions of interest (ROI) were chosen along the vessel’s lumen. G-indices were calculated for each ROI and the average of eight indices represented the G-index of a vessel.

### 4.6. Enzyme-Linked Immunosorbent Assay

To examine changes in the levels of glycocalyx components in the plasma and brain, samples were harvested from animals anesthetized with ketamine (80–100 mg/kg). For plasma isolation, blood was collected in ethylenediaminetetraacetic acid (EDTA) tubes and centrifuged at 1500× *g*, for 15 min, at 4 °C. Plasma was aliquoted in polypropylene tubes and stored at −80 °C until assays were performed. Brain tissue from the frontal cortex was excised, rinsed in ice-cold PBS (pH 7.0–7.2) to remove excess blood, snap frozen on dry ice, and stored at −80 °C. The glycocalyx levels of plasma and brain tissue were measured by commercial enzyme-linked immunosorbent assay, ELISA, kits (HA, Echelon Biosciences Inc., Salt Lake City, UT, USA; HS, CS, and SD-2, Amsbio LLC, Cambridge, MA, USA). To perform brain tissue ELISA assays, frontal cortex tissue in PBS containing 1X protease and phosphatase inhibitor cocktail was homogenized with sonication. Following this, the homogenates were centrifugated according to each manufacturer’s protocol. The supernatants were collected. Protein concentrations were determined using a Pierce BCA Protein Assay kit (Thermo Fisher Scientific, Waltham, MA, USA). Amounts of 100 µg of protein for HA and HS, 10 µg of protein for CS, and 50 µg for SD-2 were loaded and the assays were conducted following manufacturers’ instructions.

### 4.7. Surgical Preparation and Instrumentation for CBF Monitoring

Animals were anesthetized with intraperitoneal injections of acepromazine (4–6 mg/kg) and ketamine (80–100 mg/kg). Reduced doses of acepromazine (2 mg/kg) and ketamine (20–40 mg/kg) were used via intraperitoneal or intramuscular route to maintain a surgical plane of anesthesia (no response to toe pinch). Following initial induction of anesthesia, buprenorphine (0.1 mg/kg) was administered subcutaneously for additional analgesia. The femoral artery was catheterized with polyethylene (PE-50) tubing for blood sampling and measurement of blood pressure and heart rate (DataScope, Datascope Corporation, Montvale, NJ, USA). Blood samples were analyzed for pH, PaCO_2_, and PaO_2_ (partial pressure of carbon dioxide and oxygen, respectively) on a blood gas analyzer (GEM 5000, Werfen, Bedford, MA, USA). The animals were implanted with an endotracheal tube and ventilated using a small animal ventilator (MouseVent, Kent Scientific Corp., Lichtfield, CT, USA). Inspired gases were 40% medical-grade oxygen (O_2_) mixed with 60% nitrogen (N_2_) (Airgas USA, Hyattsville, MD, USA). Ventilator settings were adjusted to maintain normocapnia (PaCO_2_ of 35–45 mmHg) and normoxia (PaO_2_ ≥ 80 mmHg) [4]. CBF monitoring was performed by a laser Doppler probe (moorVMS-LDF1, Moor Instruments Inc., Wilmington, DE, USA) positioned on top of the exposed right parietal bone for a duration of 2 h. During CBF monitoring, the animals were subjected to 4 episodes of hypercapnia at intervals of 30 min, to assess the responsiveness of CBF to hypercapnia. Hypercapnia was induced by switching the ventilated gas to a mixture of 7.5% CO_2_, 40%O_2_, and 52.5% N_2_ (Airgas USA, Hyattsville, MD, USA) for 60–90 s as described by Abutarboush et al. [4]. Blood sampling was conducted before and immediately after hypercapnic exposures to determine a decrease in pH and increases in PaCO_2_ and PaO_2_ in response to inhaled mixtures of gases with higher concentrations of CO_2_ and O_2_.

### 4.8. Data Analysis

Data analyses were performed using GraphPad Prism 9.4.1 (Dotmatics, Boston, MA, USA). Data were analyzed for outliers (+/−2 standard deviations), and the necessary assumptions for parametric analysis were tested. For semi-quantitative analysis of the glycocalyx (Figure 2) and concentrations of glycocalyx components in the frontal cortex and plasma (Figure 3), a one-way analysis of variance (ANOVA) with multiple comparisons (Dunnett’s test) was used if the assumptions were met, with Welch’s test used, if necessary, based on the data’s standard deviations and variances. If the assumptions for parametric tests were not met, a Kruskal–Wallis test with Dunn’s multiple comparisons test was used instead. For analysis of CBF data (Figure 4), a mixed-effects model with repeated measures and Dunnett’s multiple comparisons test was used; if the data did not meet the assumptions for parametric methods, the aligned rank transform method was used to transform the data before the analysis.

## Figures and Tables

**Table 1:** Temporal effects of blast on glycocalyx and its components in the brain and plasma. Statistically significant results are presented. KW: Kruskal–Wallis test, with Dunn’s multiple comparisons test for comparisons of the blast groups to sham (test statistic for multiple comparisons is Z); F (DFn, DFd) indicates results from a parametric one-way ANOVA, where DF indicates the statistical degrees of freedom; W (DFn, DFd) indicates the use of Welch’s ANOVA test; EM: electron micrographs; G1: group 1; G2: group 2.

Variable	Overall *p*-Value	Overall Test Statistic	G1	G2	*p*-Value	Test Statistic
Glycocalyx Percentage (EM)	<0.0001	KW = 242.3	Sham	2 h	<0.0001	Z = 9.895
			Sham	1 d	<0.0001	Z = 9.592
			Sham	3 d	<0.0001	Z = 11.14
			Sham	14 d	<0.0001	Z = 4.476
Glycocalyx thickness (EM)	<0.0001	KW = 237.2	Sham	2 h	<0.0001	Z = 9.108
			Sham	1 d	<0.0001	Z = 9.220
			Sham	3 d	<0.0001	Z = 9.676
			Sham	14 d	0.0115	Z = 3.049
Glycocalyx G-Index (EM)	<0.0001	KW = 232.7	Sham	2 h	<0.0001	Z = 9.244
			Sham	1 d	<0.0001	Z = 9.915
			Sham	3 d	<0.0001	Z = 9.038
			Sham	14 d	0.0037	Z = 3.376
Frontal Cortex HSPG/Syndecan-2	0.0303	F (DFn, DFd) = 2.701 (6, 33)	Sham	0 h	0.0041	q (DF) = 3.710 (33)
Plasma HSPG/Syndecan-2	0.0281	F (DFn, DFd) = 2.761 (6, 32)				
Frontal Cortex Heparan Sulphate	0.0019	F (DFn, DFd) = 4.531 (6, 33)	Sham	14 d	0.0250	q (DF) = 3.004 (33)
			Sham	28 d	0.0039	q (DF) = 3.729 (33)
Frontal Cortex Chondroitin Sulphate	0.0027	W (DFn, DFd) = 6.154 (6.000, 13.63)	Sham	28 d	0.0023	t (DF) = 6.212 (7.043)
Frontal Cortex Hyaluronic Acid	0.0074	F (DFn, DFd) = 3.651 (3, 31)				
Plasma Hyaluronic Acid	<0.0001	W (DFn, DFd) = 12.75 (6.000, 31.29)	Sham	14 d	0.0201	t (DF) = 3.351 (14.82)
			Sham	28 d	0.0266	t (DF) = 3.518 (13.76)

**Table 2:** Temporal effect of blast on cerebral blood flow (CBF) and responsiveness to hypercapnia. Statistically significant results of two-way analysis of variance (ANOVA) or non-parametric equivalents are presented. The F (DFn, DFd) test statistic indicates the variable was analyzed with one-way ANOVA (using mixed-effects analysis), where DF indicates the statistical degrees of freedom;, and q represents the test statistic using Dunnett’s multiple comparisons test for comparisons of the time groups to sham (test statistic for multiple comparisons is q); G1: group 1; G2: group 2.

Variable	Factor	Overall *p*-Value	Overall Test Statistic	G1	G2	*p*-Value	Test Statistic
CBF (Flux)	Group	0.0002	F (DFn, DFd) = 6.818 (5, 31)	Sham	2 h	<0.0001	q (DF) = 13.74 (65.86)
				Sham	1 d	<0.0001	q (DF) = 14.58 (65.56)
				Sham	3 d	0.0003	q (DF) = 4.376 (58.91)
				Sham	14 d	<0.0001	q (DF) = 8.291 (60.64)
				Sham	28 d	<0.0001	q (DF) = 18.86 (75.77)
	Time	0.0321	F (DFn, DFd) = 2.658 (4.183, 129.2)	0 min	90 min	0.0255	q (DF) = 3.070 (36.00)
	Interaction	0.0021	F (DFn, DFd) = 1.878 (40, 247)				
∆CBF with Hypercapnia	Group	0.0204	F (DFn, DFd) = 3.156 (5, 31)	Sham	2 h	<0.0001	q (DF) = 6.165 (44.72)
				Sham	1 d	<0.0001	q (DF) = 6.130 (44.90)
				Sham	3 d	0.0440	q (DF) = 2.667 (40.10)
				Sham	14 d	0.0021	q (DF) = 3.860 (35.97)
				Sham	28 d	<0.0001	q (DF) = 7.760 (48.89)
Recovery Time	Group	<0.0001	F (DFn, DFd) = 8.311 (5, 31)	Sham	2 h	0.0014	q (DF) = 3.923 (44.88)
				Sham	3 d	<0.0001	q (DF) = 6.328 (37.89)

## Data Availability

Data are contained within the article.
